# Impact of CPAP Therapy Adherence on Global Cognition in Patients with Moderate to Severe Obstructive Sleep Apnea: A One-Year Follow-Up

**DOI:** 10.3390/medicina59050846

**Published:** 2023-04-27

**Authors:** Diana Raluca Velescu, Monica Steluta Marc, Camelia Corina Pescaru, Daniel Traila, Emanuela Vaștag, Ion Papava, Alexandru Catalin Motofelea, Ioana Mihaiela Ciuca, Diana Manolescu, Cristian Oancea

**Affiliations:** 1 Pulmonary Department, Center for Research and Innovation in Precision Medicine of Respiratory Diseases (CRIPMRD), ‘Victor Babes’ University of Medicine and Pharmacy, 300041 Timisoara, Romania; velescu.diana@umft.ro (D.R.V.); pescaru.camelia@umft.ro (C.C.P.); emanuela.tudorache@umft.ro (E.V.); dmanolescu@umft.ro (D.M.); oancea@umft.ro (C.O.); 2 Clinical Hospital of Infectious Diseases and Pneumophthisiology ‘Dr. Victor Babes’, 300173 Timisoara, Romania; 3 Neuroscience Department, NEUROPSY—COG Center for Cognitive Research in Neuropsychiatric Pathology, ‘Victor Babes’, University of Medicine and Pharmacy, 300041 Timisoara, Romania; papava.ion@umft.ro; 4 Internal Medicine Department, ‘Victor Babes’ University of Medicine and Pharmacy Timisoara, 2 Eftimie Murgu Sq, 300041 Timisoara, Romania; alexandru.motofelea@umft.ro; 5 Pediatric Department, ‘Victor Babes’ University of Medicine and Pharmacy Timisoara, Eftimie Murgu Square No. 2, 300041 Timisoara, Romania; ciuca.ioana@umft.ro; 6 Radiology and Medical Imaging Department, ‘Victor Babes’ University of Medicine and Pharmacy Timisoara, Eftimie Murgu Square No. 2, 300041 Timisoara, Romania

**Keywords:** cognition, obstructive sleep apnea, continuous positive airway pressure, adherence

## Abstract

*Background:* Obstructive sleep apnea increases (OSA) cognitive impairment risk. The objective of this study was to determine the impact of continuous positive airway pressure (CPAP) adherence on global cognition using the Montreal Cognitive Assessment questionnaire (MoCA). *Materials and Methods:* Thirty-four new patients diagnosed with moderate or severe OSA (apnea-hypopnea index AHI ≥ 15 events/h) from the CPAP group were compared with thirty-one moderate to severe OSA patients from the no-CPAP group. In addition, all patients completed the MoCA test, a patient health questionnaire (PHQ-9) for depressive symptoms, and a generalized anxiety questionnaire (GAD-7) for anxiety symptoms, at baseline, after 6 months, and after 1 year. *Results:* At baseline, there were no significant differences between the two groups regarding total MoCA scores, 20.9 ± 3.5 CPAP group to 19.7 ± 2.9 no-CPAP group (*p* = 0.159), PHQ-9 (*p* = 0.651), and GAD-7 (*p* = 0.691). After one year, improvement was observed for a total MoCA score of 22.7 ± 3.5 (*p* < 0.001) for the CPAP group, and significant variance of score between groups was more accentuated for delayed recall and attention (*p* < 0.001) sub-topic. Moreover, PHQ-9, GAD-7 scores, and the Epworth Sleepiness Scale (ESS) decreased significantly (*p* < 0.001) after CPAP therapy. The MoCA score was significantly correlated with years of education (r = 0.74, *p* < 0.001) and had a negative correlation with body mass index (BMI) (r = −0.34), ESS (r = −0.30) and PHQ-9 (r = −0.34). *Conclusions:* One year of CPAP adherence improved global cognition associated with OSA.

## 1. Introduction

Sleep-related breathing disorders are a significant medical and social problem. The most common subtype is obstructive sleep apnea (OSA), which is characterized by disruptions to sleep architecture that can cause excessive daytime sleepiness, increased cardiovascular risk [1], metabolic disorders [2], cognitive impairment and decline [3], and affective changes [4]. Symptoms of OSA include snoring, apnea episodes, choking episodes, sleep fragmentation, insomnia, morning headaches, excessive daytime sleepiness, irritability, poor concentration, decreased memory, and loss of libido [5].

According to a global estimate, 936 million people aged 30–69 suffer from obstructive sleep apnea, defined as having five or more obstructive respiratory events per hour [6]. These numbers change periodically, along with the increase in obesity among the population. Patients with OSA manifest a decline in a wide spectrum of cognitive functions, including memory, attention, executive, verbal and visuospatial skills, and psychomotor speed [7]. Evidence suggests the association between OSA and cognitive impairment, but the mechanisms are complex and unclear. Intermittent hypoxia and sleep fragmentation contribute to subsequent oxidative stress, inflammation, reperfusion injury, and endothelial dysfunction leading to brain alteration, especially in brain regions responsible for cognitive and emotional functions [3,8].

CPAP is the gold standard treatment for OSA, which prevents upper airway collapse during sleep and improves mortality with long use [9]. Effective CPAP therapy improves oxygenation due to reducing nighttime breathing events, daytime sleepiness, sleep-related quality of life, and mood alteration such as depression and anxiety [10,11]. The data from the literature have shown that therapy not only reduces daytime and nocturnal symptoms but also improves cognitive functions [12]. Studies involving magnetic resonance imaging of the brain studying untreated OSA patients identified atrophy of the neocortex and cerebellum, decreased volume of the hippocampal dentate gyrus, and prefrontal atrophy in the severe form of OSA [13]. Moreover, Castronovo et al. showed a decrease in white matter fiber integrity in OSA patients using diffusion tensor imaging. After 12 months of CPAP therapy, improvements were observed in all affected regions, suggesting that some brain abnormalities can be recovered with effective treatment [14,15]. These conclusions indicated the importance of early diagnosis and treatment for patients with OSA to prevent cognitive decline. 

The present study aimed to investigate the long-term efficacy of CPAP therapy adherence for moderate to severe OSA patients on global cognition and psychological conditions compared with the scores of a control group consisting of patients who refused the treatment.

## 2. Materials and Methods

This study was conducted as a prospective observational study. The patients in this study were recruited from the Clinical Hospital of Infectious Diseases and Pneumophtisiology “Dr. Victor Babes” Timisoara. Of 103 participants, 65 consecutive eligible participants meeting inclusion criteria were invited to participate. They were divided into two groups: thirty-four in the CPAP group and thirty-one in the no-CPAP group. The study followed participants for 6 months and 1 year, asking them to complete questionnaires. All subjects in this study were volunteers who signed a written informed consent, which the local ethics committee of the Clinical Hospital of Infectious Diseases and Pneumophtisiology “Dr. Victor Babes” Timisoara approved (No. 3546/2022). The informed consent detailed the procedures of the study as well as the possible risks and benefits. It also guaranteed that the subjects could end their participation at any time.

The exclusion criteria for the current study were: (1) age below 45 years; (2) AHI < 15 events/h; (3) history of central nervous system disease, including cerebrovascular accident, neurodegenerative disease, epilepsy, and brain tumors; (4) alcohol and drug abuse; (5) patients who had formerly undergone CPAP therapy and those who discontinued CPAP treatment during the study or no adherence patients (<4 h/night and at least 70% of the time during a month); (6) those unable to complete the questionnaires meaningfully; (7) patients with acute or chronic respiratory failure, malignant lung diseases, pneumothorax; (8) those with severe chronic disease (unstable angina pectoris, recent myocardial infarction, unstable heart failure, chronic kidney insufficiency, liver insufficiency, inflammatory disease, and oncologic patients).

Smoking status, anthropometric data such as age, sex, height, weight, body mass index (BMI), neck and abdomen circumference, and comorbidities (essential hypertension, ischemic heart disease, atrial fibrillation, heart failure, diabetes mellitus, dyslipidemia, COPD, and asthma) were collected from all the subjects.

All patients were monitored overnight using a cardiorespiratory polygraphy system (Sleep Doc Porti 7, Version 5.19b, Hechingen, Germany), which recorded data on oxygen desaturation, breathing, respiratory movements, and snoring. Apnea (cessation of airflow for 10 s) and hypopnea (decrease in amplitude with 50 to 90% airflow reduction from baseline, associated with >3% oxygen desaturation or arousal) were defined according to the American Academy of Sleep Medicine criteria from 1999 [16]. Apnea Hypopnea Index ≥ 15 events/h means moderate to severe OSA. The amount of time spent during sleep with an arterial oxygen saturation level below 90% indicated hypoxemia (t90). Before performing the polygraphy, patients were instructed to avoid alcohol and daytime sleeping.

### 2.1. Neuropsychological Assessment

All eligible patients with OSA from the two groups completed the neuropsychological assessment at baseline after six months and one-year follow-up, including MoCA, ESS, PHQ-9, and GAD-7 scales. MoCA is a 10-min screening tool with high specificity and sensitivity for detecting early cognitive impairment. It evaluates several cognitive domains such as short-term memory recall, visual-spatial, and executive function, attention, concentration, working memory, language, and orientation in time and space to differentiate healthy cognitive aging from mild cognitive impairment [17]. From the maximum score of 30 points, a meta-analysis concluded that the MoCA cutoff score for cognitive impairment was 23, and this value has been found to have excellent sensitivity and specificity (0.96 and 0.95) [18].

The Epworth Sleepiness Scale (ESS) was used to assess daytime sleepiness. The questionnaire includes eight passive or active specific daily situations, and patients were asked to indicate the tendency to fall asleep on a 0–3 scale, with 0 meaning no chance of falling asleep and 3 showing a high chance of falling asleep. The total score is 24, where a score of 10 points and more means clinical levels of excessive daytime sleepiness. The current study used the standardized Romanian test version [19].

Screening for depressive distress, as well as the grade of depressive symptoms severity, was performed with the Patient Health Questionnaire-9 Depression Scale, a self-reported validated questionnaire used by primary care practitioners [20]. The scale consists of nine questions assessing the patient’s health status during the previous two weeks. It is related to sadness, tiredness, sleepiness or sleeping too much, little interest in doing things, thoughts of personal failure, poor concentration, low self-confidence, slow or fast speech, and suicidal ideation. A total score ranges from 0 to 27, and a cut-off value ≥ 10 was used to indicate clinically significant depressive symptoms [21].

The severity of anxiety was measured with the Generalized Anxiety Disorder Questionnaire-7 Scale. It includes seven questions about feeling nervous, on edge, unable to control worrying, being worried about different things, inability to relax, being very restless so that it is hard to sit, getting irritated quickly, and feeling afraid that something horrible might happen. Moderate anxiety symptoms were present with a cut-off point over 10 from 27 total scores [22].

### 2.2. CPAP Therapy

All thirty-four OSA patients from the CPAP group received therapy for at least one year with compliance ≥4 h/night and at least 70% of the time during a month. Adherence at home was objectively reported by reading the sim card from the machine every three months, including duration of use and AHI, mask leakage, and pressure in auto adjustment mode.

### 2.3. Statistical Analysis

Continuous data are presented as a mean with SD where data are normally distributed and as a median with the 25th and 75th centiles for non-parametric data. Categorical data are summarized as frequencies and percentages. Differences between groups for continuous normally distributed data were tested using Welch’s *t*-test for two groups or ANOVA when there were more than two groups and post hoc tests were conducted using the Bonferroni correction to adjust for multiple comparisons when appropriate. Non-parametric continuous data were tested using a Mann–Whitney U test for two groups or the Kruskal–Wallis test for three or more groups. Differences across categorical data were tested using the χ^2^ test or Fisher’s exact test when expected cell counts were less than five. All statistical analyses were performed with R (version 3.6.3) using the tidyverse, finalfit, mcgv, survival, stringdist, janitor, and Hmisc packages.

## 3. Results

Baseline demographic data, polygraphy measurements, comorbidities, and questionnaire scores between the groups are related in Table 1. Thirty-four patients from the CPAP group comprised 18 men (52.9%) and 16 women (47.1%). The no-CPAP group included 17 men (54.8%) and 14 women (45.2%). According to the AHI classification, 24.61% of participants had a moderate form of OSA (AHI = 15–29.9 events/h), and 75.38% had a severe form (AHI ≥ 30 events/h).

OSA patients are associated with comorbidities, and for this study, there were in order of frequency: arterial hypertension (98.5%), dyslipidemia (89.2%), ischemic heart disease (49.2%), heart failure (47.7%), diabetes (44.6%), asthma (24.6%), COPD (18.5%), and arrhythmias (16.9%).

There were no significant differences between the groups at baseline according to total MoCA score (*p* = 0.159), PHQ-9 (*p* = 0.651), and GAD-7 (*p* = 0.691). In our study, for the MoCA score, a result of <23 points is relevant for cognitive impairment, and 70.76% of study participants meet this criterion, 22 (64.70%) in the CPAP group and 24 (77.41%) in the no-CPAP group.

The stratification of characteristics and questionnaires of the study population based on sex showed similar values between males and females, but we observed that men had higher values regarding Table 2. The average BMI for women was 37.3 ± 7 compared with 36.2 ± 5.6 in men with no significance (*p* = 0.438). According to a PHQ-9 score > 10, which indicates clinical depression, we observed that females (63.3%) tend to be more depressed than men (40%). Clinical anxiety (GAD-7 score > 10) was 34.3% for men and 23% for women.

The MoCA score was significantly correlated with years of education (r = 0.74, *p* < 0.0001) and had a negative correlation with body mass index (BMI) (r = −0.345, *p* < 0.005), ESS (r = −0.304, *p* = 0.014), and PHQ-9 (r = −0.341, *p* < 0.005). On the one hand, regarding AHI, we observed a strong correlation with ODI (t = 0.76, *p* < 0.0001) and a moderate correlation with ESS (r = 0.44, *p* < 0.0001), and affective change GAD-7 score (r = 0.42, *p* < 0.0001), and PHQ-9 (r = 0.40, *p* < 0.0001). On the other hand, there was a positive correlation between the ESS and the two affective scales, PHQ-9 (r = 0.395, *p* < 0.001) and GAD-7 (r = 0.304, *p* = 0.014), which indicates that reported daytime sleepiness is associate with depressive and anxiety symptoms.

**Figure 1:**
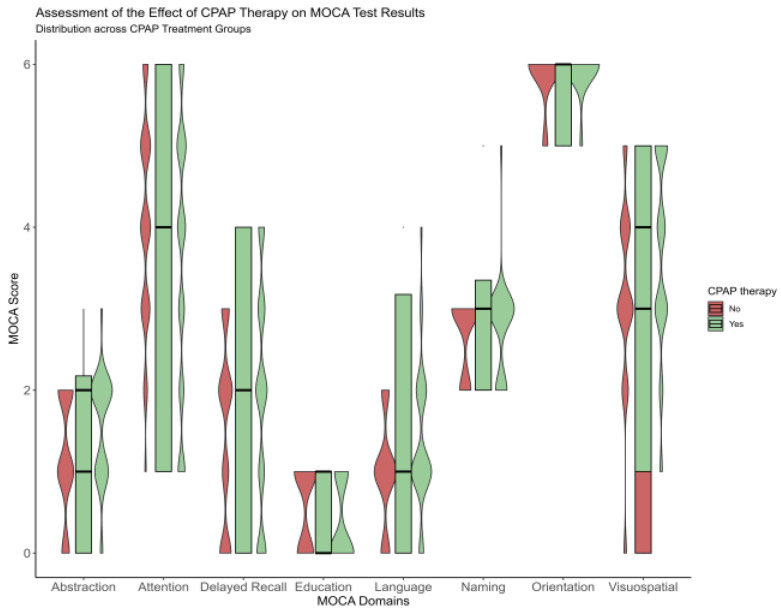
Violinplot showing the difference between the no-CPAP group and CPAP group after a one-year follow-up, according to Moca domains.

After one year, improvement was observed for a total MoCA score from 20.9 ± 3.5 to 22.7 ± 3.5 for the CPAP group, and significant score variance between groups was more accentuated for delayed recall and attention (*p* < 0.001) sub-topic Figure 1. At baseline, for the CPAP group, the delayed recall was 1.9 ± 1.4, and attention was 3.6 ± 1.7. However, after one year of follow-up, these domains improved significantly, such as 2.9 ± 1 for delayed recall and 4.1 ± 12 for attention, respectively, Figure 1 and Table A1.

**Figure 2:**
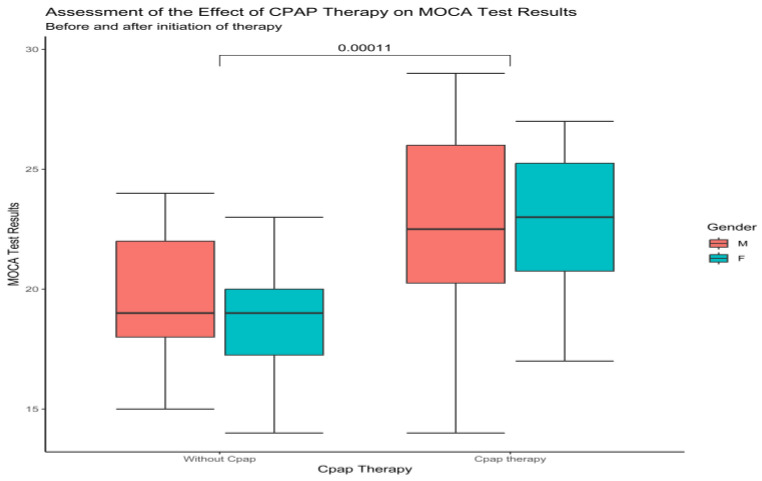
Boxplot showing the difference between males and females at baseline and after CPAP therapy according to MoCa (Wilcoxon test).

Upon examination of the total MoCa score between males and females, at baseline and one-year follow-up, in the no-CPAP group, the results showed 20 ± 2.8 to 20 ± 2.81 for males and 19 ± 3 to 19 ± 2.6 for females. There was no significant improvement or decrease in the total Moca score for this group. Compared to this group, the results from the CPAP group showed significant improvement (*p* < 0.0001) for males, 22.1 ± 4 to 23 ± 3.8, and 21 ± 3.4 to 22.6 ± 3 for females (Figure 2).

Our results showed that after adjusting for confounders, OSA therapy was not significantly associated with MoCA score at 1 year (*p* = 0.175). However, OSA severity at 6 months was positively associated with the MoCA score at 1 year (*p* = 0.004), while OSA severity at 1 year was negatively associated with the MoCA score at 1 year (*p* = 0.002). These findings suggest that the relationship between OSA severity and cognitive function may be dynamic and change over time, highlighting the importance of longitudinal monitoring and management of OSA (Table 3).

Moreover, PHQ-9, GAD-7 scores and the Epworth Sleepiness Scale (ESS) decreased significantly (*p* < 0.001) after CPAP therapy (Table 4). Only one woman and three men manifested clinical depression symptoms after one year of treatment. In the CPAP group, after 6 months of therapy, AHI decreased from 48 ± 15.8 to 4.3 ± 2.4, and after 1 year, to 3.5 ± 2.

## 4. Discussion

The present study aimed to evaluate the impact of CPAP therapy adherence on global cognition in patients with moderate to severe OSA over one year and to compare the neuropsychological tests with an untreated OSA group. Patients with sleep breathing disorders have a clear and noticeable difficulty with brain function. CPAP is an excellent therapy option for patients with sleep apnea who frequently report daytime tiredness, memory loss, and attention deficit [12,23]. We found that long CPAP therapy improves total MoCA score and significant results regarding domain as delayed recall and attention. Several studies have been published in the current literature regarding brain functional change for OSA patients. However, the mechanism underlying the relationship still needs to be fully understood. Results from other studies suggested that sleep fragmentation and oxygen desaturation could be involved in cognitive impairment. The recurrent cessation of nocturnal breathing causes repeated arousal during sleep and leads to excessive daytime somnolence. According to a review study, sleep disturbance and daytime sleepiness affected people’s ability to pay attention, be alert, learn, and remember things. In addition, hypoxia has also been shown to be a significant predictor of executive dysfunction and frontal impairment [23,24]. In contrast with these findings, we found a negative correlation between the total MoCA score and ESS (r = −0.304, *p* = 0.014) and with t90 (r = −0.265, *p* = 0.033).

Obesity is the strongest risk factor for OSA and has a predictive role in cognitive impairment. Our result showed a negative correlation between the total MoCA score and BMI (r = −0.345, *p* < 0.005). Previous studies have examined the association and relationship of obesity markers, such as BMI, fat mass, abdominal fat, and waist-to-hip ratio, with cognitive impairment [3,25,26]. According to Polsek et al. [27], comorbid obesity may accelerate the evolution of AD in people with OSA because of its strong association with OSA and its potential role in neurodegenerative processes [27]. Adipocytes release pro-inflammatory cytokines that change synaptic and neural plasticity, contributing to neurodegenerative processes, which explains an elevated risk. Sun et al. [28] evaluated the cognitive status of OSAS patients using MoCA, Mini-Mental State Examination (MMSE), and ESS. Deficiencies were observed in visual space, attention, executive function, and delayed recall. In addition, the OSA severe group had elevated levels of leptin, high sensitive-Protein C Reactive (hs-CRP), and tumor necrosis factor (TNF-α). After six months of CPAP therapy, both inflammation and cognitive impairment improved.

Untreated OSA is associated with comorbidities and an increased risk of developing severe medical conditions [1]. For example, in our study population, hypertension (98.5%) and dyslipidemia (89.2%) were more frequent for this lot. In addition, half of the participants suffered from ischemic heart disease, compensated heart failure, and diabetes. A recent study that involved 1440 participants concluded that systolic hypertension in midlife and its persistence into later life was linked to a 1.6- to 2-fold increase in dementia risk at an 18-year follow-up [29]. Several possible paths that connect hypertension pressure to dementia were described, such as small vessel disease, atherosclerosis of the major arteries, and cardiac dysfunction that predisposes to cerebral hypoperfusion [29]. CPAP therapy improves blood pressure, especially in those OSA patients with resistant hypertension [30]. In a recent pilot trial, people with MCI who adhered to CPAP treatment for a year showed significant gains in psychomotor/cognitive processing speed compared to subjects with MCI and OSA who did not adhere to CPAP treatment [31]. However, both groups had similar rates of hypertension, like in our study participants. Additionally, research has connected dementia to specific cardiovascular conditions such as coronary heart disease, atrial fibrillation, and heart failure [32]. Given the chain reaction of unfavorable repercussions caused by both OSA and cardiovascular illness, treating OSA may help attenuate its harmful effects on brain health. A meta-analysis of 2.3 million people with type 2 diabetes concluded that these participants had a 1.6-fold increase in the risk of developing dementia [33].

The results of our study showed that 18.5% of OSA participants suffer from COPD. Ten out of twelve patients had a severe OSA form (AHI > 30 event/h) and a low MoCA total score of 18 ± 2.01. According to the research population and neuropsychological testing, the prevalence of cognitive impairment in COPD patients ranges from 12% to 88% [34]. The association of the two diseases, OSA and COPD, may increase the risk of cognitive impairment.

The severity of sleep apnea, daytime sleepiness, and mood alteration such as depression and anxiety improved after one year of treatment (*p* < 0.001) compared to the no-CPAP group. The PHQ-9 score was previously demonstrated to be a responsive measure of depression symptoms in moderate to severe OSA patients receiving 3 months of CPAP therapy [35]. At baseline, there was a strong correlation between the PHQ-9 score and AHI, ODI, and ESS. A review conducted by Kemer and Roose developed a model that investigates how cerebral hypoperfusion, endothelial dysfunction, and neuroinflammation associated with OSA could start or exacerbate the onset of cerebral small vessel disease and blood–brain barrier dysfunction, conduct in white matter lesions, gray matter loss, abnormalities in the white matter fiber tract, neuronal damage, synaptic plasticity, and neurodegenerative processes, which leads to depressive symptoms and cognitive impairment [36]. In contrast with our findings, Dostálová et al. conducted a study of OSA patients and followed the short-term effect of 3 months of CPAP therapy on cognition using the MoCA questionnaire, depression using the Beck Depression Inventory, and the Stare-Trait Inventory for anxiety, and they found a decrease in scores for daytime sleepiness, depression, and anxiety. However, there was no improvement in cognitive performance because there was no control group and a lack of cognitive impairment before starting therapy [37]. The dates of anxiety in OSA are less prevalent than depression but not uncommon. We found that clinical anxiety (GAD-7 score > 10) was 34.3% for men and 23% for women for our study, but the total score GAD-7 had no significant correlation with the total MoCa score (*p* = 0.035).

In our study group, 38% of participants were current smokers or ex-smokers, with smoking 27.2 ± 6.3 packages/per year. A Chinese study that included current smokers, ex-smokers, and non-smokers aged 20–60 concluded that the association between OSA and chronic smoking leads to cognitive impairment than smoking alone [38]. Moreover, a meta-analysis that included 37 studies found that smokers have a high risk for dementia, and smoking cessation decreases this risk [39].

We found a strong correlation between the MoCa score and years of education (r = 0.742, *p* < 0.0001), and this result showed that years of education have an impact on performance in the majority of MoCA domains. A recent study demonstrated that education had a relatively small impact on the memory-recall domain [40]. After one year of CPAP therapy, the delayed recall domain (1.9 ± 1.4 to 2.9 ± 1) and attention (3.6 ± 1.7 to 4.1 ± 1.2) improved for our study.

Despite the advantages of CPAP therapy on global cognition, excessive daytime sleepiness, depression, and anxiety, patients encounter difficulties when using it, such as financial, social, and psychological issues. Only 41% of diagnosed patients adhere to treatment after one year [41]. The governments do not support the high cost of treatment in many countries, and a patient cannot afford to start the treatment. This situation led to significant financial pressure on health care. For example, Joshua M. Bock et al. found that the annual care cost of adherent CPAP patients with cardiovascular comorbidities was lower than those of the non-adherent group ($6825, $11,312; *p* < 0.05). The healthcare investment was reduced by 40% in one year [42]. Data from the literature showed that mandibular advancement devices are an economical alternative treatment to evaluate responder or nonresponder OSA patients [43]. A recent study concluded that multiple combined factors are responsible for cognitive impairment, such as age, genetics, socioeconomic factors, nutrition, and physical activity [44]. Therefore, it is necessary to have a complete approach to the treatment and prevention of cognitive impairment.

### Study’s Limitations

Some limitations remain in this study. First, the small number of participants and lack of adjustment for confounders were not racially, ethnically, or geographically diverse. Second, the study’s average year of education was 12 ± 3.3, which may explain the lower results in a MoCA total score. Moreover, the study used one instrument for assessing cognitive impairment. However, the MoCa has been validated widely to evaluate cognitive impairment and cognitive decline. Third, this study was not randomized, so patients who use CPAP may be better about other health factors that may impact cognition. These limitations indicate that a bigger diverse group of patients is necessary to support better study findings.

## 5. Conclusions

Obstructive sleep apnea is a prevalent condition with a significant impact on global cognition and across cognitive domains. CPAP therapy improves global cognition over a one-year follow-up and cognitive domain, especially delayed recall and attention. Moreover, therapy improved excessive daytime sleepiness and depressive and anxiety symptoms. The highly prevalent comorbidities associated with OSA and cognitive impairment indicate that the recovery of patients involves teamwork and an interdisciplinary approach.

## Figures and Tables

**Table 1:** Description of study groups, comorbidities, and scores at baseline.

	Total (n = 65)	No-CPAP (n = 31)	CPAP (n = 34)	*p*-Value
Age (years)	58.9 (45.0, 78,0)	60.4 (47.0, 78.0)	57.6 (45.0, 78.0)	0.196
Sex, male (%)	35 (53.8)	17 (54.8)	18 (52.9)	0.878
BMI	36.7 (21.4, 50.4)	37.2 (24.6, 51.2)	36.2 (21.4, 50.4)	0.522
Neck circumference (cm)	41.6 (36.0, 52.0)	40.8 (36.0, 46.0)	42.3 (36.0, 52.0)	0.052
Abdomen circumference (cm)	126.4 (107.0, 154.0)	125.8 (111.0, 137.0)	127.0 (107.0, 154.0)	0.618
Smoking status, n (%)	25 (38.5)	14 (45.2)	11 (32.4)	0.289
Pack-years	27.2 (15.0, 40.0)	25.5 (15.0, 35.0)	29.3 (20.0, 40.0)	0.138
Comorbidities, n (%)				
Arterial hypertension	64 (98.5)	30 (96.8)	34 (100)	0.291
Dyslipidemia	58 (89.2)	27 (87.1)	31 (91.2)	0.599
Heart failure	31 (47.7)	16 (51.6)	15 (44.1)	0.546
Ischemic heart disease	32 (49.2)	16 (51.6)	16 (47.1)	0.714
Arrhythmias	11 (16.9)	5 (16.1)	6 (17.6)	0.870
Diabetes	29 (44.6)	15 (48.4)	14 (41.2)	0.559
Asthma	16 (24.6)	8 (25.8)	8 (23.5)	0.831
COPD	12 (18.5)	6 (19.4)	6 (17.6)	0.859
AHI	40.4 (16.6, 79.7)	32 (15.6, 77.7)	48 (18.3, 97.7)	<0.001
ODI	44.4 (15.1, 89.1)	38.5 (15.1, 73.9)	49.9 (21.6, 89.1)	0.002
t90	31.4 (0.7, 86.9)	30.5 (0.7, 86.9)	32.3 (1.3, 85.0)	0.782
Years of education	12 (6.0, 18.0)	10.9 (6.0, 16.0)	13.1 (6.0, 18.0)	0.005
MoCA	20.3 (14.0, 26.0)	19.7 (14.0, 25.0)	20.9 (14.0, 26.0)	0.159
ESS	13.9 (6.0, 24.0)	12.7 (6.0, 21.0)	14.9 (6.0, 24.0)	0.043
PHQ-9 score	11.2 (3.0, 25.0)	10.9 (3.0, 22.0)	11.5 (3.0, 25.0)	0.651
GAD-7 score	8.9 (2.0, 21.0)	8.6 (2.0, 19.0)	9.1 (2.0, 21.0)	0.691

Results are present as median and interquartile range; BMI, body mass index; AHI, apnea-hypopnea index; ODI, oxygen desaturation index; t90, total sleep time spent at an arterial oxygen saturation <90%; MoCa, Montreal Cognitive Assessment questionnaire; ESS, Epworth sleepiness scale; PHQ-9, patient health questionnaire; GAD-7, generalized anxiety disorder questionnaire; CPAP, continuous positive airway pressure.

**Table 2:** Clinical characteristics at baseline stratified by sex.

		Male (n = 35)	Female (n = 30)	Total (n = 65)	*p*-Value
**Age**	Median (IQR)	59.0 (53.5 to 64.5)	57.0 (49.0 to 64.8)	58.0 (52.0 to 65.0)	0.497
**BMI**	Median (IQR)	35.5 (32.9 to 40.3)	38.0 (32.2 to 41.0)	37.2 (32.3 to 40.9)	0.438
**Neck (cm)**	Median (IQR)	42.0 (41.0 to 45.5)	39.0 (38.0 to 41.0)	41.0 (39.0 to 43.0)	<0.001
**Abdomen (cm)**	Median (IQR)	128.0 (123.0 to 132.0)	127.0 (118.2 to 130.0)	128.0 (120.0 to 132.0)	0.233
**Smoking**	No	19 (54.3)	21 (70.0)	40 (61.5)	0.297
	Yes	16 (45.7)	9 (30.0)	25 (38.5)	
**Pack-years**	Median (IQR)	30.0 (20.0 to 30.0)	26.0 (23.8 to 30.5)	30.0 (20.0 to 30.0)	0.929
**AHI**	Median (IQR)	40.3 (27.9 to 53.7)	37.0 (28.1 to 51.5)	37.5 (28.1 to 52.6)	0.567
**ODI**	Median (IQR)	43.3 (33.6 to 55.2)	41.0 (34.1 to 54.6)	41.8 (33.6 to 55.5)	0.708
**ESS**	Median (IQR)	15.0 (9.5 to 17.5)	13.0 (11.2 to 15.8)	13.0 (10.0 to 17.0)	0.625
**PHQ9 score**	Median (IQR)	10.0 (7.0 to 14.0)	11.0 (8.2 to 13.0)	11.0 (8.0 to 14.0)	0.259
	n (%)	21 (60.0)	11 (36.7)	32 (49.2)	0.104
**GAD-7 score**	Median (IQR)	7.0 (5.0 to 12.0)	9.0 (7.0 to 10.0)	8.0 (5.0 to 12.0)	0.395
	n (%)	23 (65.7)	23 (76.7)	46 (70.8)	0.487
**MoCa total score**	Median (IQR)	20.0 (18.0 to 23.0)	20.0 (18.0 to 22.8)	20.0 (18.0 to 23.0)	0.583

Results are presented as median and interquartile range; BMI, body mass index; AHI, apnea-hypopnea index; ODI, oxygen desaturation index; t90, total sleep time spent at an arterial oxygen saturation <90%; MoCa, Montreal Cognitive Assessment questionnaire; ESS, Epworth sleepiness scale; PHQ-9, patient health questionnaire; GAD-7, generalized anxiety disorder questionnaire.

**Table 3:** Model Coefficients—Moca score evaluated at 1 year.

Predictor	Estimate	SE	t	*p*
**Intercept**	**25.33682**	**1.66932**	15.178	<0.001
AHI	−0.04953	0.03566	−1.389	0.175
AHI at 6 months	1.04783	0.33450	3.133	0.004
Ahi at 1 year	−1.34628	0.40295	−3.341	0.002

**Table 4:** Questionnaire at baseline and follow-up.

	Total (n = 65)	No-CPAP (n = 31)	CPAP (n = 34)	*p*-Value
Baseline				
MoCA	20.3 (14, 26)	19.7 (14, 25)	20.9 (14, 26)	0.159
ESS	13.9 (6, 24)	12.7 (6, 21)	14.9 (6, 24)	0.043
PHQ-9	11.2 (3, 25)	10.9 (3, 22)	11.5 (3, 25)	0.651
GAD-7	8.9 (2, 21)	8.6 (2, 19)	9.1 (2, 21)	0.691
After 6 months				
MoCA	21 (12, 28)	19.6 (14, 25)	22.2 (12, 28)	0.003
ESS	9.7 (1, 20)	12.4 (5, 20)	7.2(1, 12)	<0.001
PHQ-9	9.3 (2, 20)	10.8 (4, 20)	7.9 (2, 16)	0.004
GAD-7	6.7 (1, 17)	7.9 (2, 17)	5.6 (1, 17)	0.006
After 1 year				
MoCA	21.1 (14, 29)	19.3 (14, 24)	22.7 (14, 29)	<0.001
ESS	7.9 (1, 20)	12.6 (6, 20)	3.7 (1, 7)	<0.001
PHQ-9	7.7 (1, 17)	9.8 (4, 17)	5.8 (1, 11)	<0.001
GAD-7	5.6 (1, 15)	7.6 (2, 15)	3.8 (1, 10)	<0.001

Results are presented as median and interquartile range; MoCa, Montreal Cognitive Assessment questionnaire; ESS, Epworth sleepiness scale; PHQ-9, patient health questionnaire; GAD-7, generalized anxiety disorder questionnaire; CPAP, continuous positive airway pressure.

## Data Availability

The data presented in this study are available on reasonable request from the corresponding author.

## Appendix A

**Table A1:** MoCa domains at baseline and follow-up.

	No-CPAP Group	CPAP Group	*p*-Value
Beseline MoCA domains			
Visuospatial	3.1 (0.0, 5.0)	3.6 (1.0, 5.0)	0.069
Naming	2.7 (2.0, 3.0)	2.8 (2.0, 5.0)	0.684
Attention	4.0 (1.0, 6.0)	3.6 (1.0, 6.0)	0.245
Language	1.0 (0.0, 2.0)	1.4( 0.0, 4.0)	0.052
Abstractisation	1.2 (0.0, 2.0)	1.6 (0.0, 3.0)	0.041
Delayed Recall	1.5 (0.0, 3.0)	1.9 (0.0, 4.0)	0.204
Orientation	5.9 (5.0, 6.0)	5.9. (3.0, 6.0)	0.336
MoCa domains after 6 months			
Visuospatial	3.1 (0.0, 5.0)	3.9 (2.0, 5.0)	0.004
Naming	2.7 (2.0, 3.0)	2.7 (2.0, 3.0)	0.615
Attention	3.4 (1.0, 6.0)	4.0 (1.0, 6.0)	0.111
Language	1.2 (0.0, 3.0)	1.6 ( 0.0, 3.0)	0.060
Abstractisation	1.2 (0.0, 2.0)	1.6 (0.0, 3.0)	0.031
Delayed Recall	1.5 (0.0, 3.0)	2.6 (1.0, 5.0)	<0.001
Orientation	5.9 (5.0, 6.0)	5.9 (3.0, 6.0)	0.849
MoCa domains after one year			
Visuospatial	3.1 (0.0, 5.0)	3.8 (1.0, 5.0)	0.017
Naming	2.7 (2.0, 3.0)	2.7 (1.0, 4.0)	0.994
Attention	3.1 (1.0, 5.0)	4.1 (2.0, 6.0)	<0.001
Language	1.3 (0.0, 3.0)	1.7 (0.0, 3.0)	0.045
Abstractisation	1.2 (0.0, 2.0)	1.6 (0.0, 3.0)	0.031
Delayed recall	1.5 (0.0, 3.0)	2.9 (1.0, 5.0)	<0.001
Orientation	5.9 (5.0, 6.0)	5.0 (3.0, 6.0)	0.617
